# Supplementary figures and images for: LFA-1 interaction with GBP-130 on Plasmodium falciparum-infected red blood cells mediates NK cell activation and parasite control
Source: eLife. 2026 May 28;15:RP110942. doi: 10.7554/eLife.110942 (PMC13218722; doi:10.7554/eLife.110942)

Figure 1B (i)

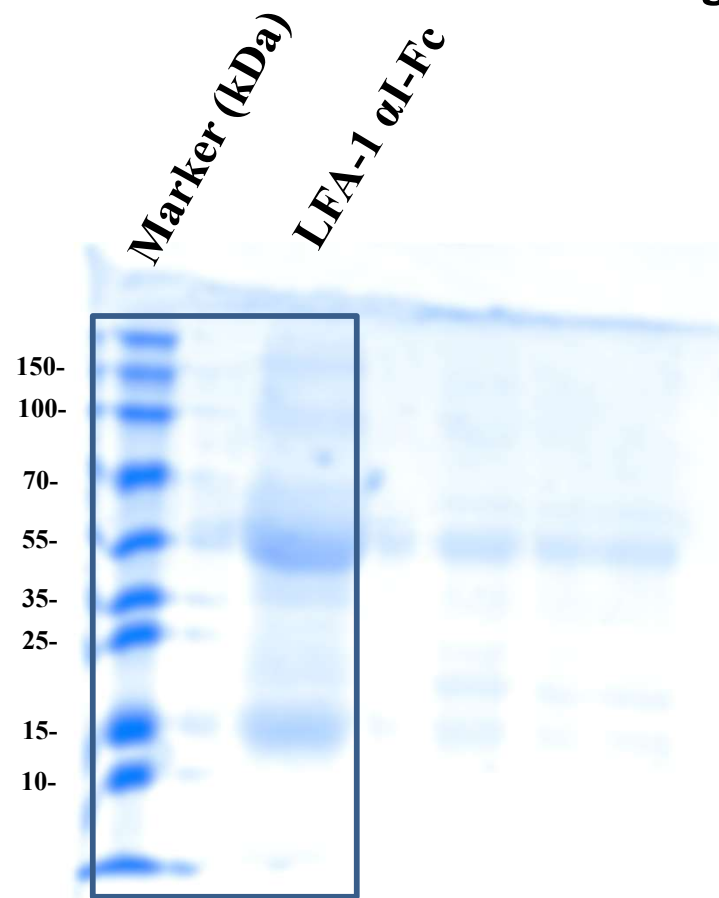

Figure 1B (ii)

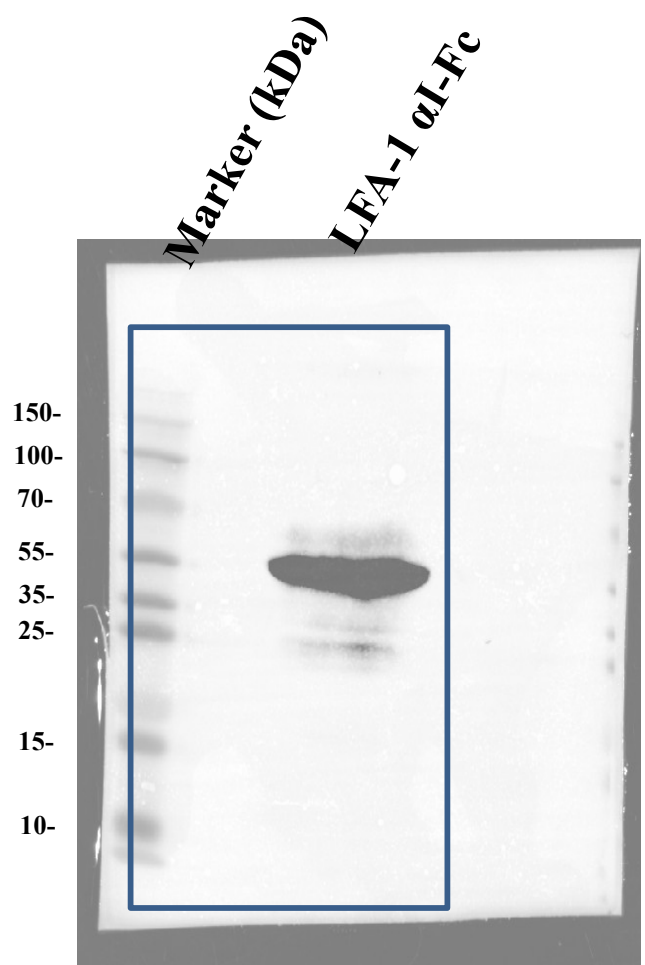

Supplement: Figure 1—source data 1. [file elife-110942-fig1-data1.zip › Figure 1-Source data 1/PDF of Raw images Figure 1B.pdf]

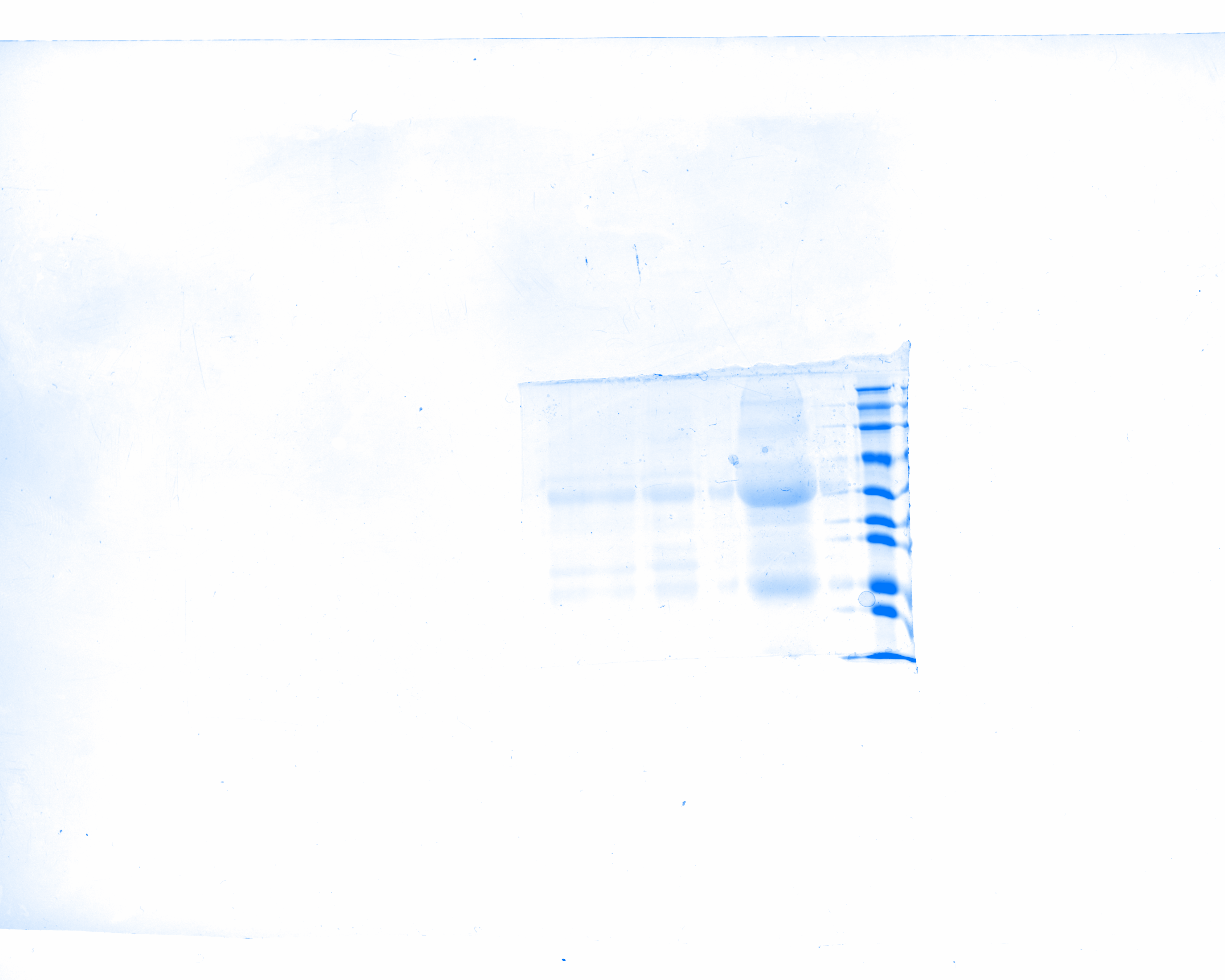

Supplement: Figure 1—source data 2. [file elife-110942-fig1-data2.zip › Figure 1-Source data 2/Figure 1 B_i.tif]

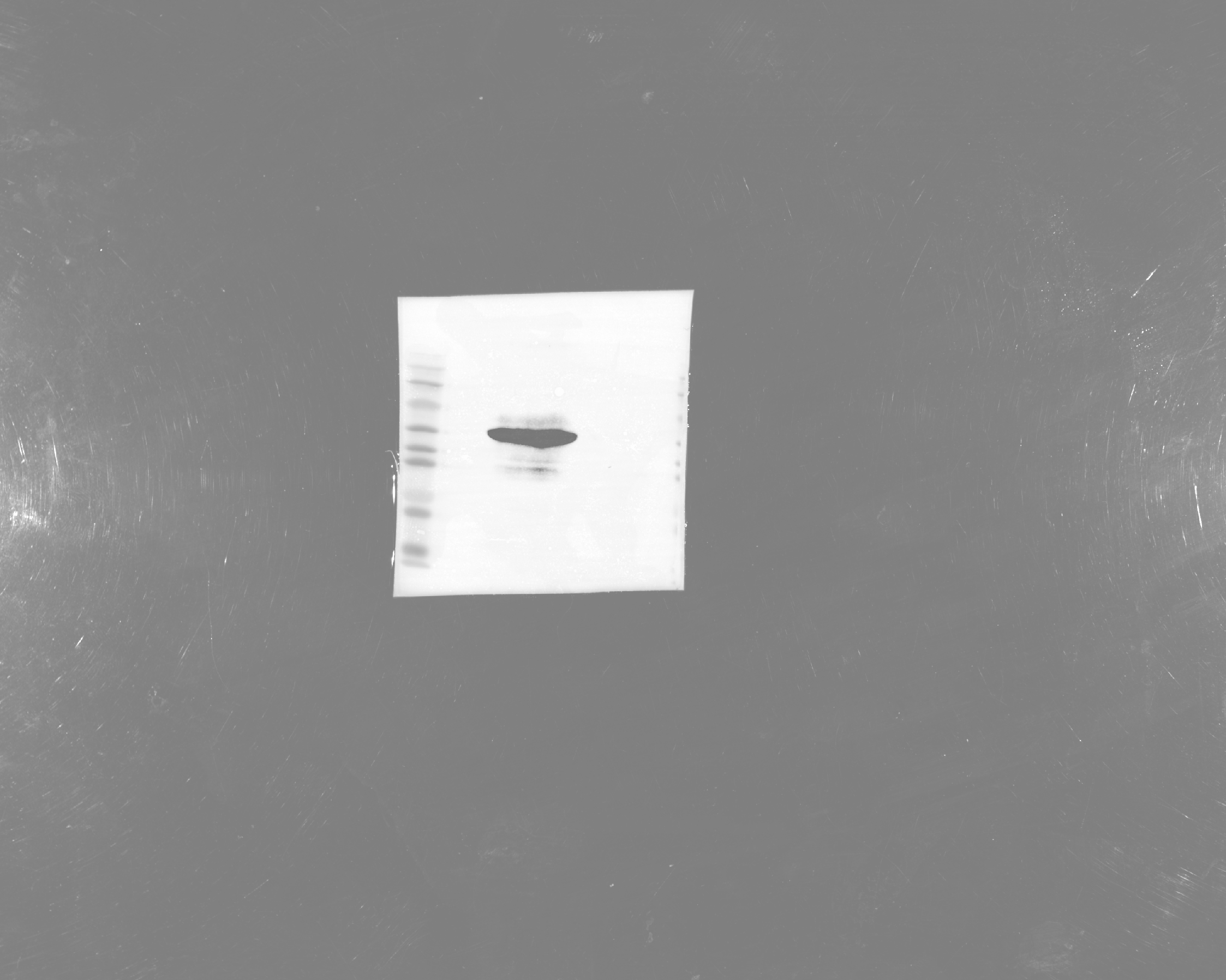

Supplement: Figure 1—source data 2. [file elife-110942-fig1-data2.zip › Figure 1-Source data 2/Figure 1B_ii.tif]

Figure 2B (ii)

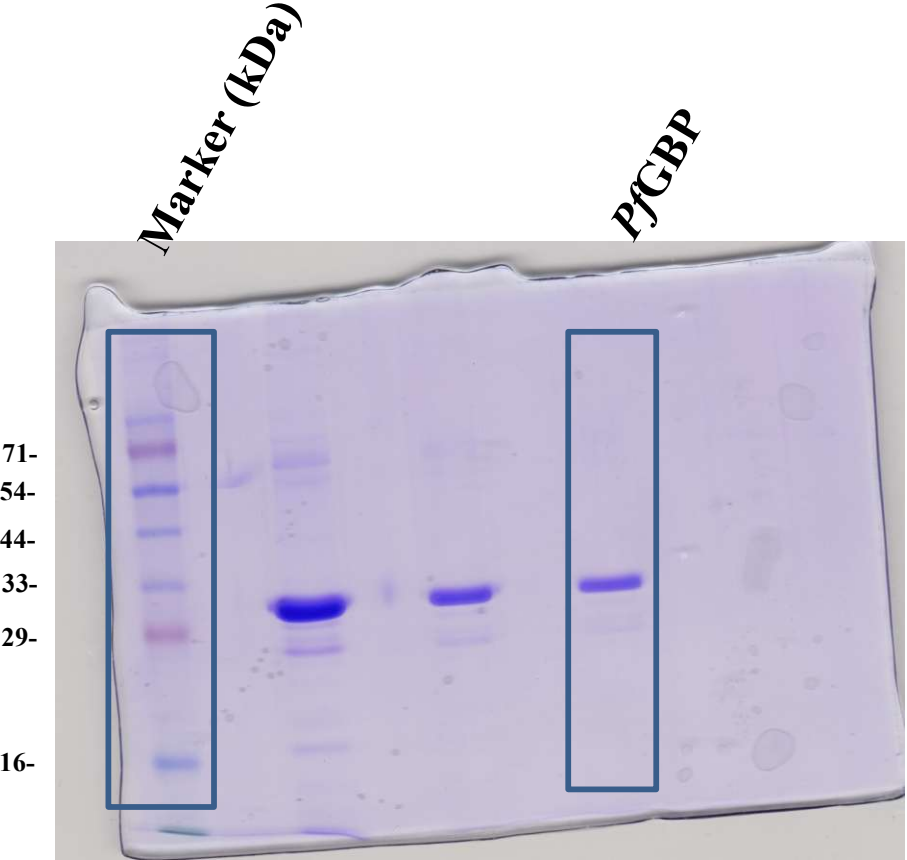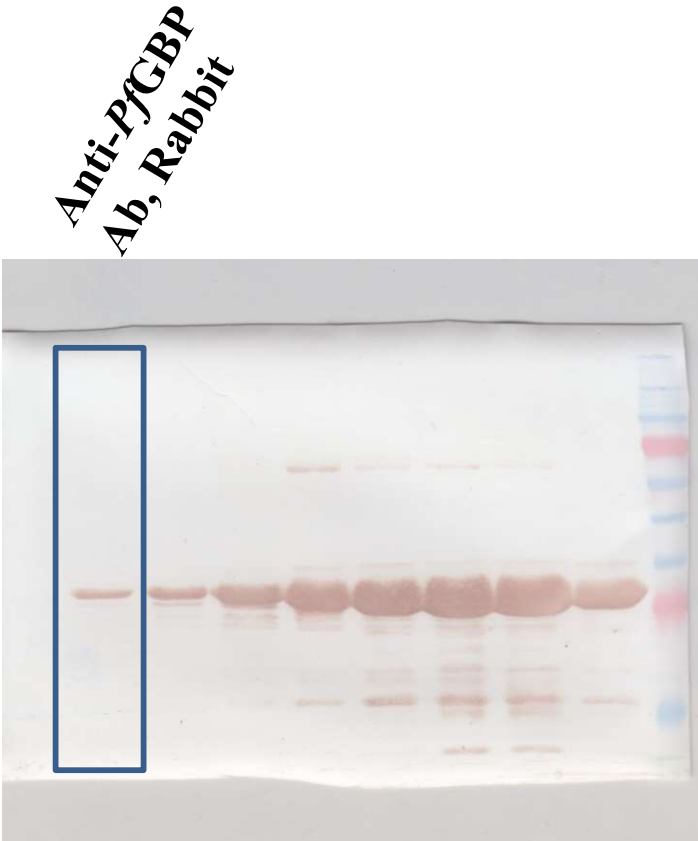

Figure 2B (iv)

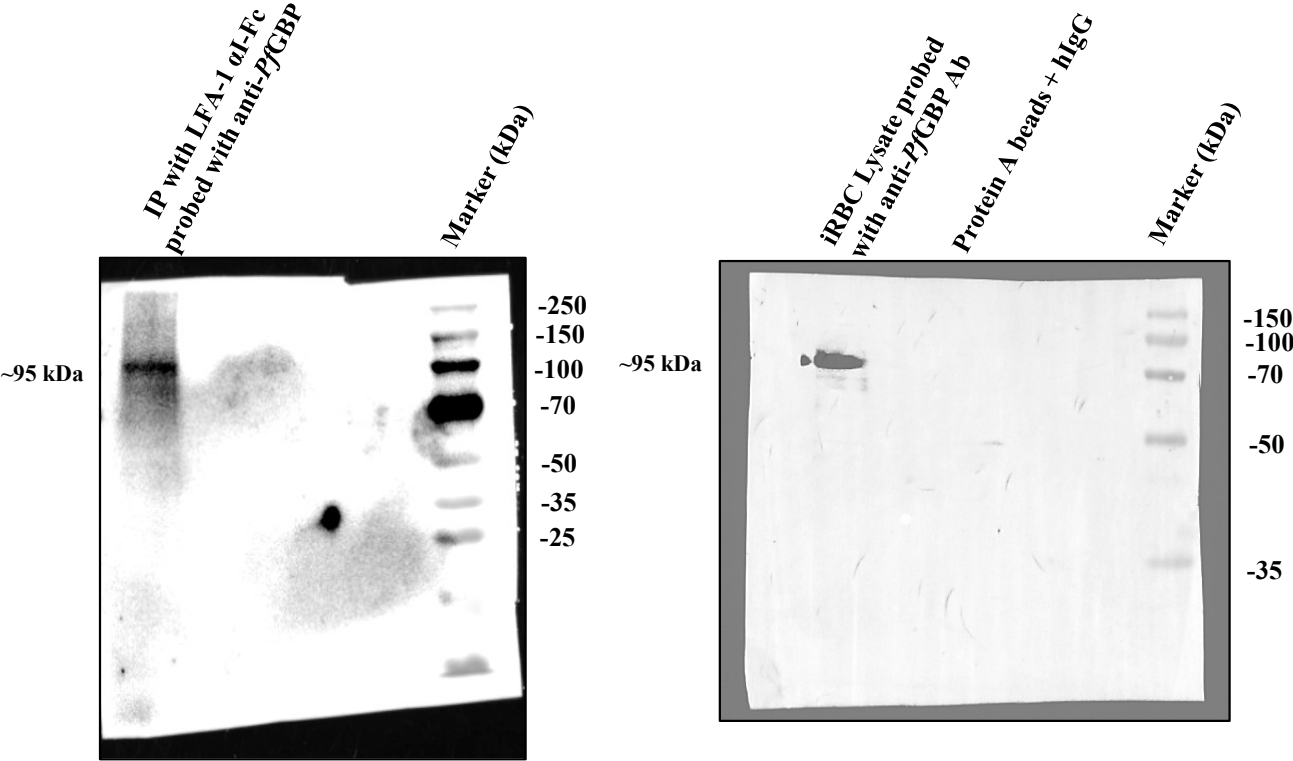

Supplement: Figure 2—source data 1. [file elife-110942-fig2-data1.zip › Figure 2-Source data 1/PDF of Raw images Figure 2B ii & iV.pdf]

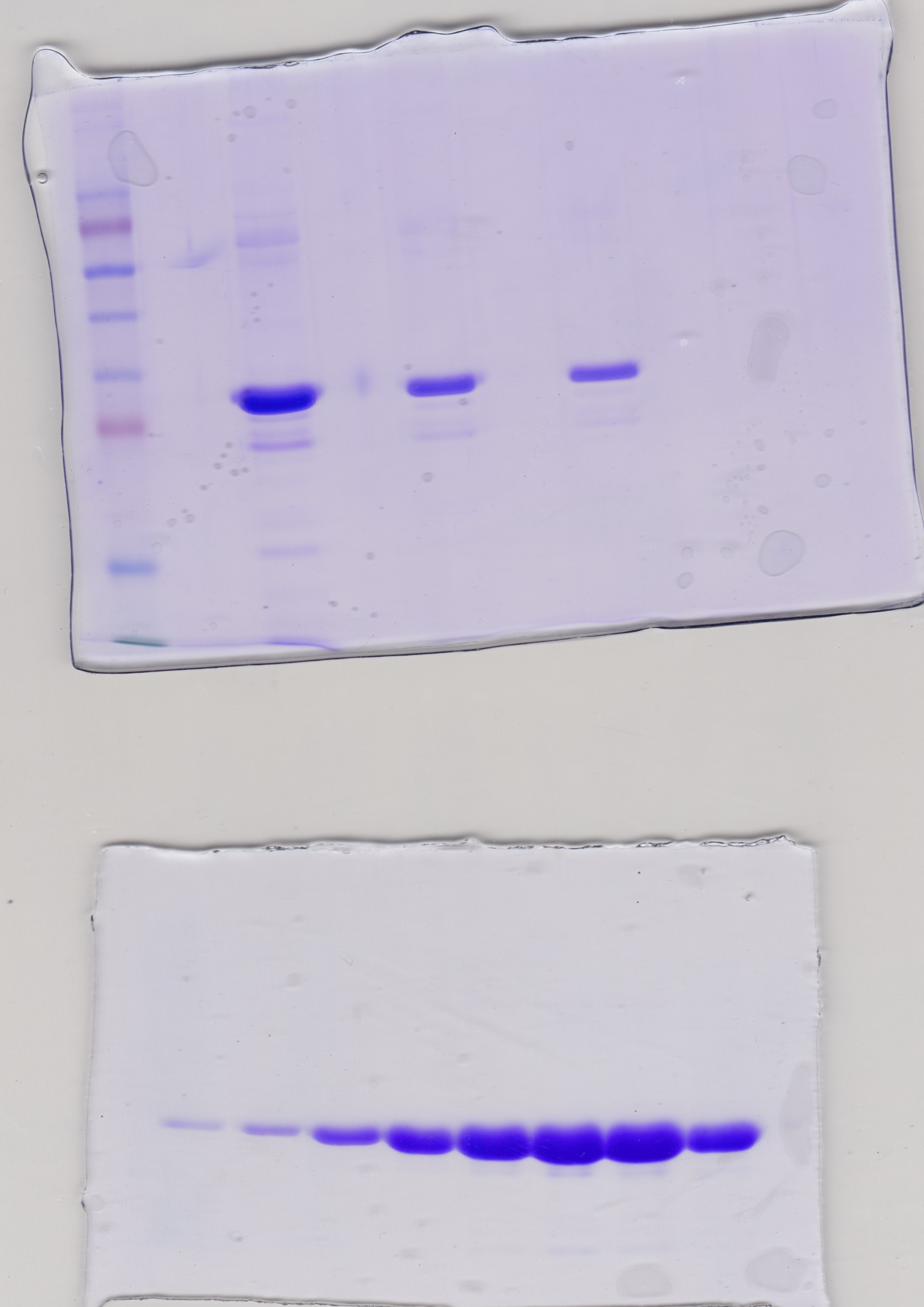

Supplement: Figure 2—source data 2. [file elife-110942-fig2-data2.zip › Figure 2-Source data 2/Figure 2B ii.jpeg]

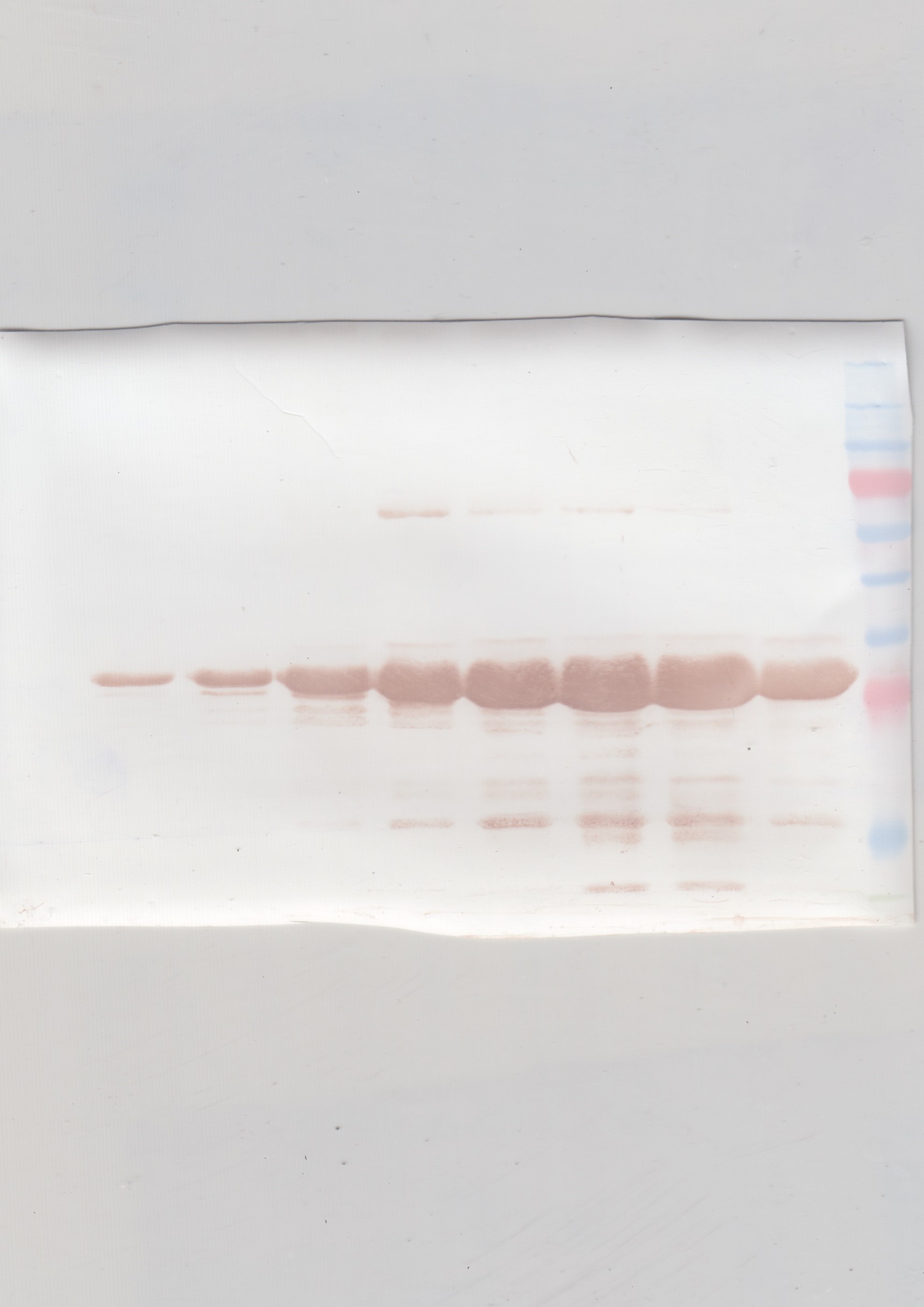

Supplement: Figure 2—source data 2. [file elife-110942-fig2-data2.zip › Figure 2-Source data 2/Figure 2B ii_Western.jpeg]

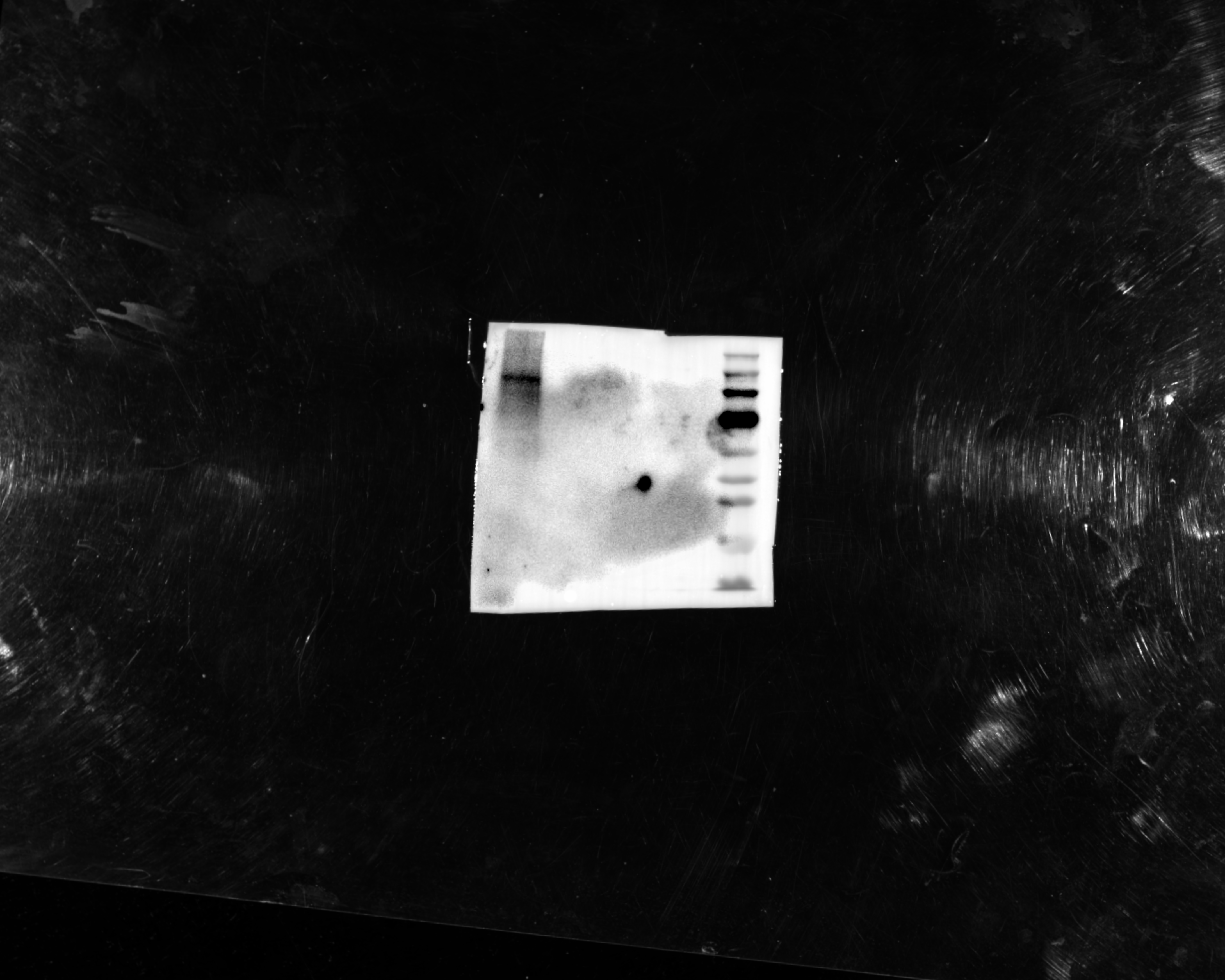

Supplement: Figure 2—source data 2. [file elife-110942-fig2-data2.zip › Figure 2-Source data 2/Figure 2B iv_IP and Bead control.tif]

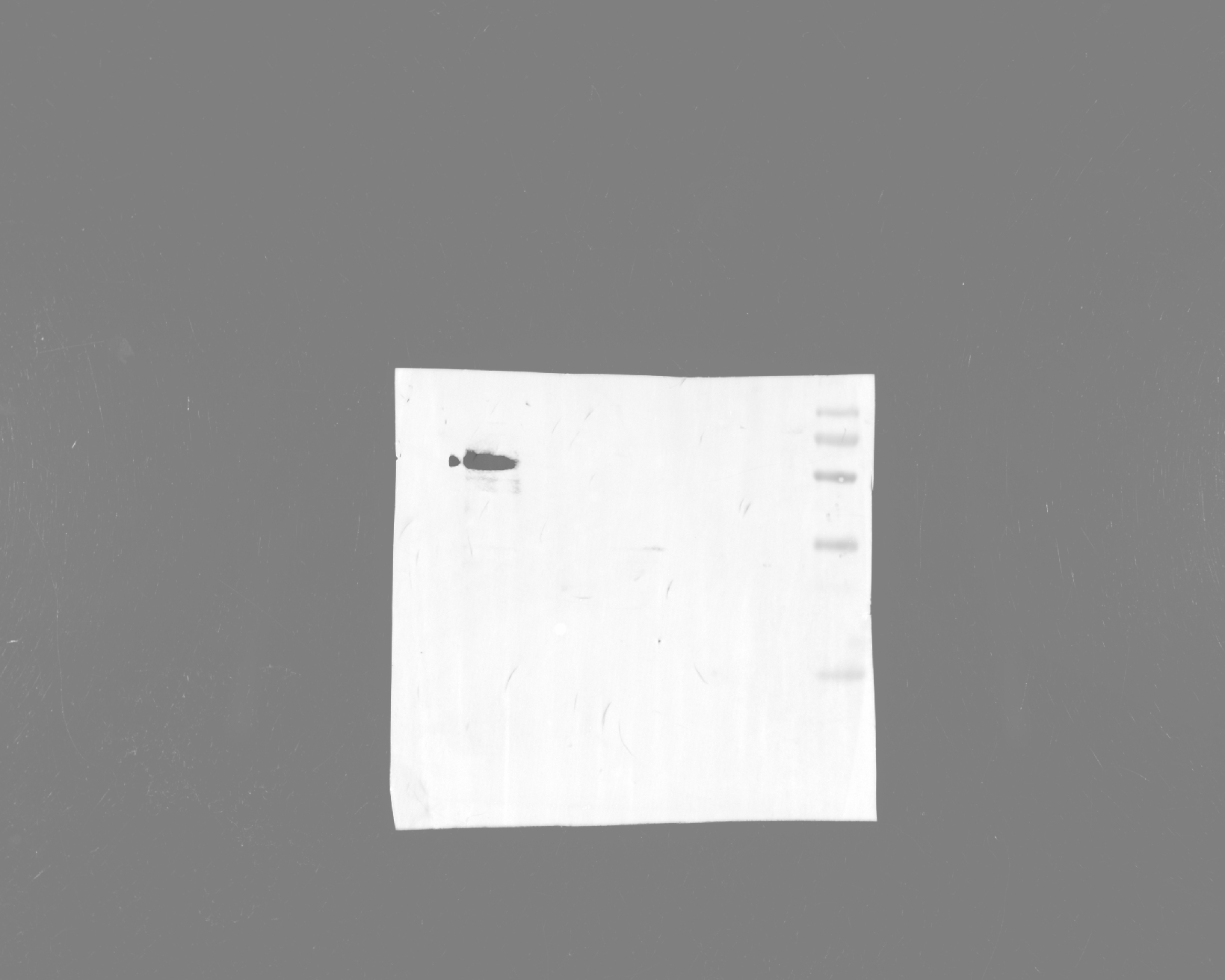

Supplement: Figure 2—source data 2. [file elife-110942-fig2-data2.zip › Figure 2-Source data 2/Figure 2B iv_iRBC Lysate.jpg]

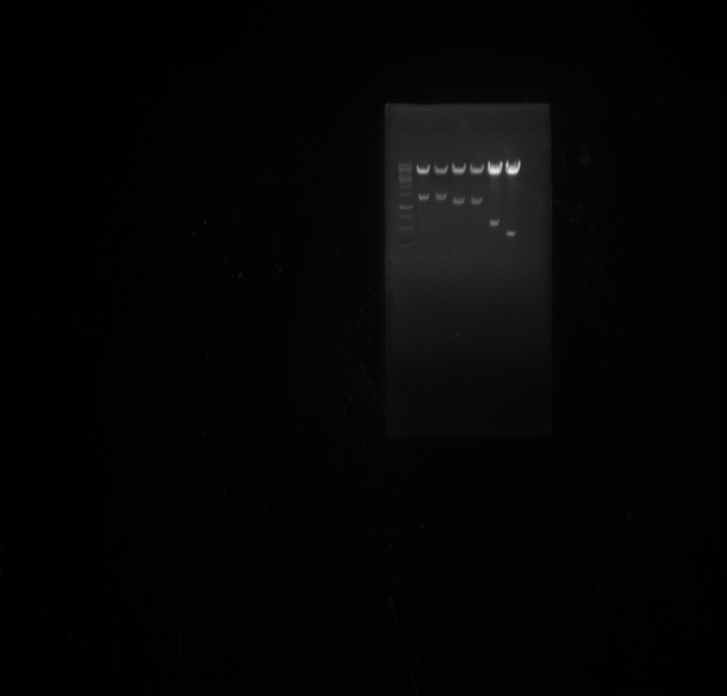

Supplement: Figure 2—figure supplement 1—source data 2. [file elife-110942-fig2-figsupp1-data2.zip › Figure 2-figure supplement 1-Source data 2/Figure 2-figure supplement 1B (Pfuse plasmid + LFA1 i-Domain Insert).tif]

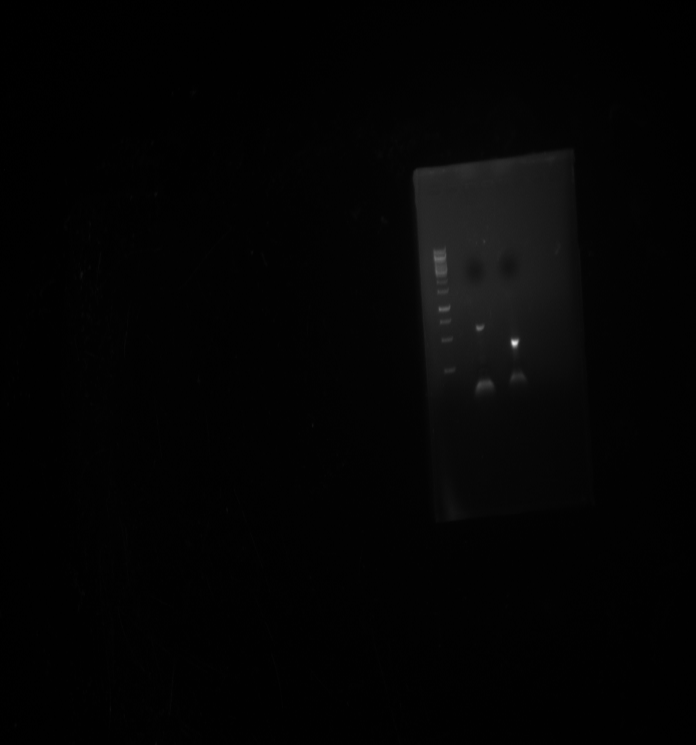

Supplement: Figure 2—figure supplement 1—source data 2. [file elife-110942-fig2-figsupp1-data2.zip › Figure 2-figure supplement 1-Source data 2/Figure 2-figure supplement 1B.tif]

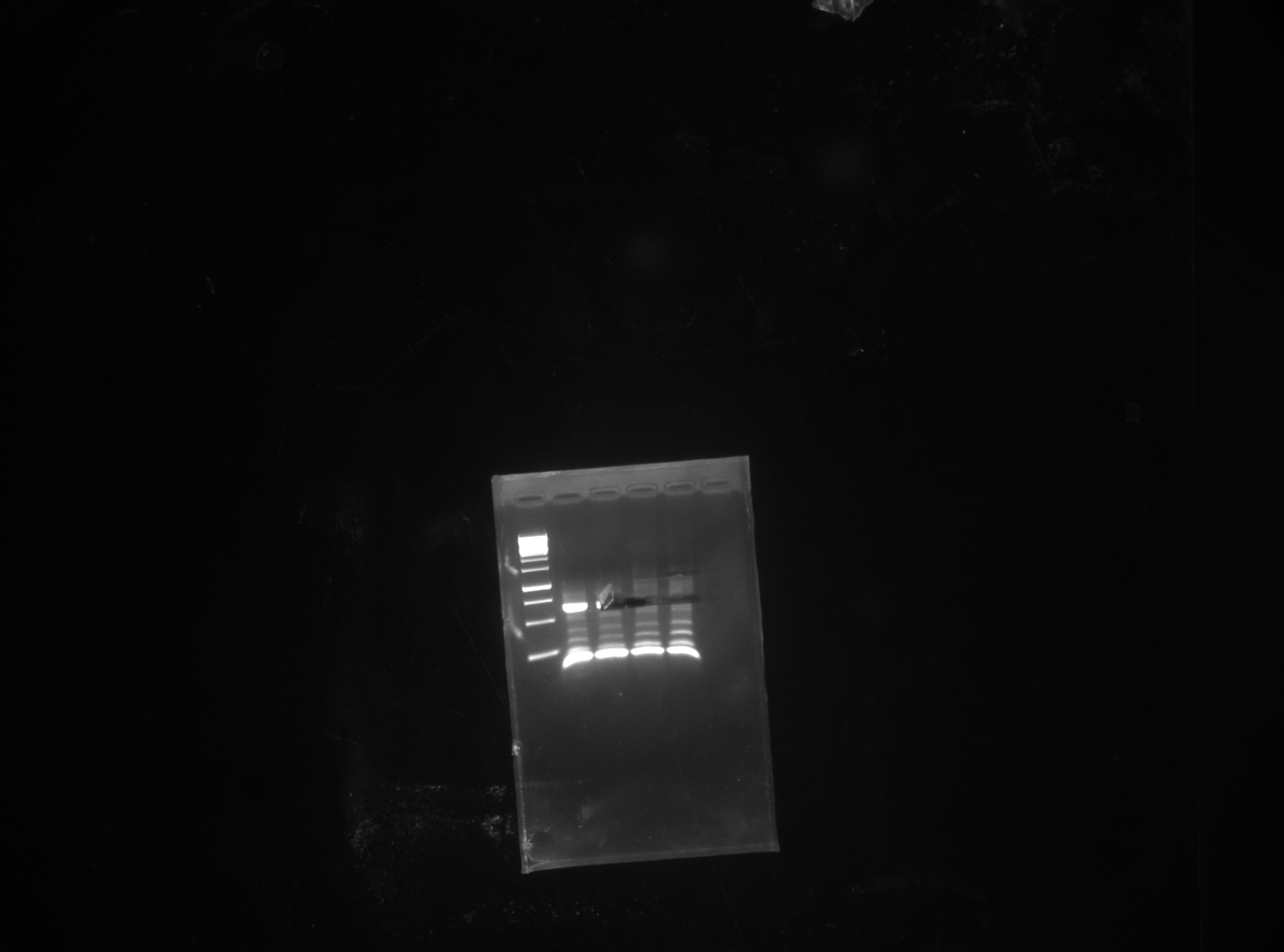

Supplement: Figure 2—figure supplement 1—source data 2. [file elife-110942-fig2-figsupp1-data2.zip › Figure 2-figure supplement 1-Source data 2/Figure 2-figure supplement 1C_i.tif]

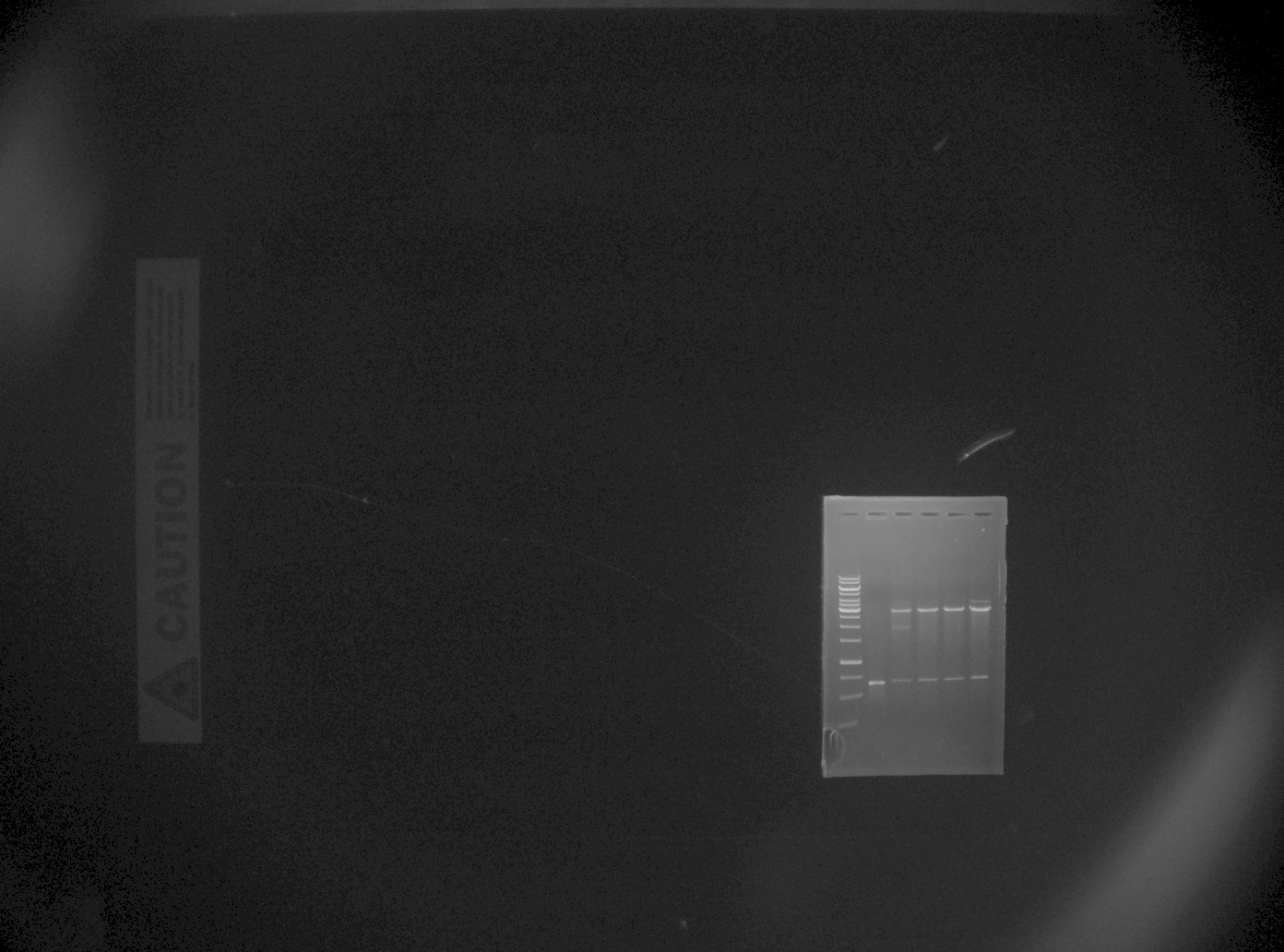

Supplement: Figure 2—figure supplement 1—source data 2. [file elife-110942-fig2-figsupp1-data2.zip › Figure 2-figure supplement 1-Source data 2/Figure 2-figure supplement 1C_ii.jpg]

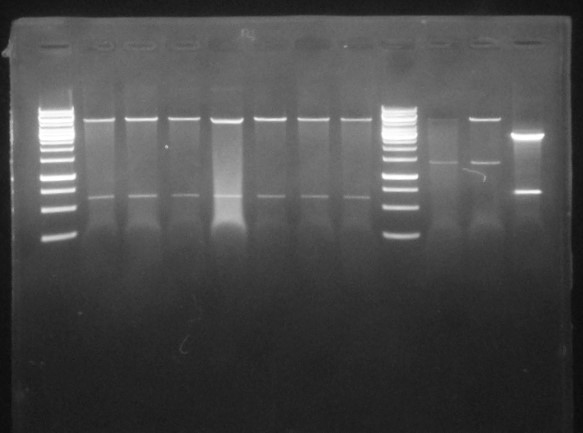

Supplement: Figure 2—figure supplement 1—source data 2. [file elife-110942-fig2-figsupp1-data2.zip › Figure 2-figure supplement 1-Source data 2/Figure 2-figure supplement 1C_iii.jpg]

Figure 3B (ii)

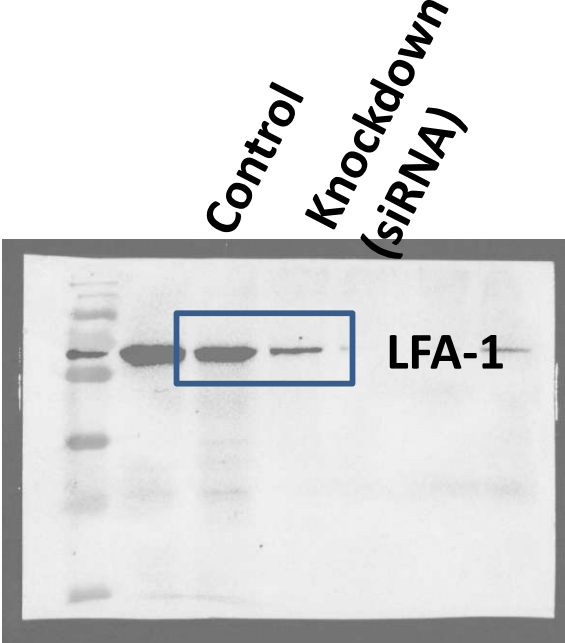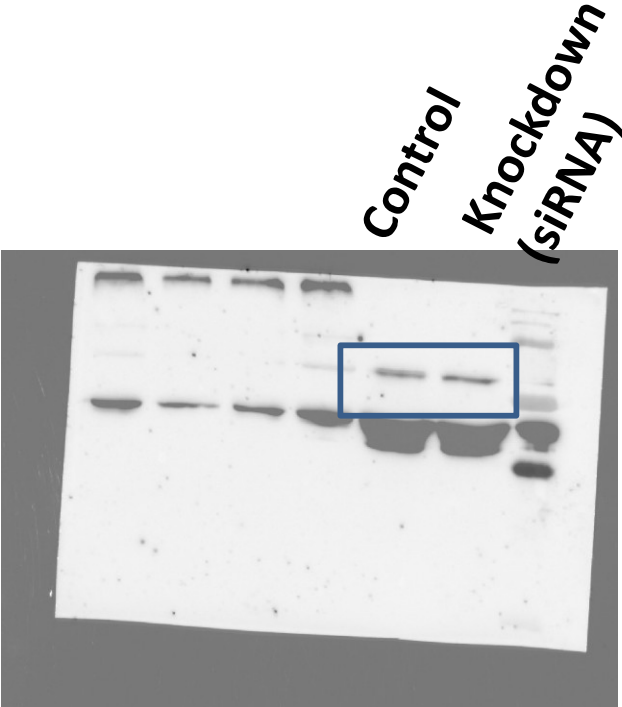

2 loading controls  
were used

$\beta$  Actin  
Anti-H2B

Figure 3C (ii)

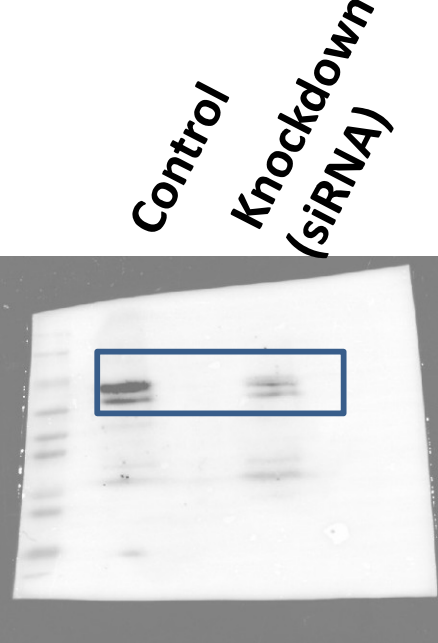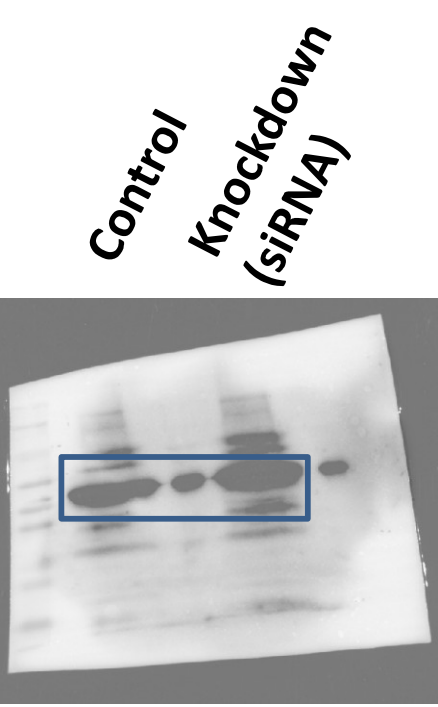

LFA-1

$\beta$  Actin

Supplement: Figure 3—source data 1. [file elife-110942-fig3-data1.zip › Figure 3-Source data 1/PDF of Raw images Figure 3B & 3C.pdf]

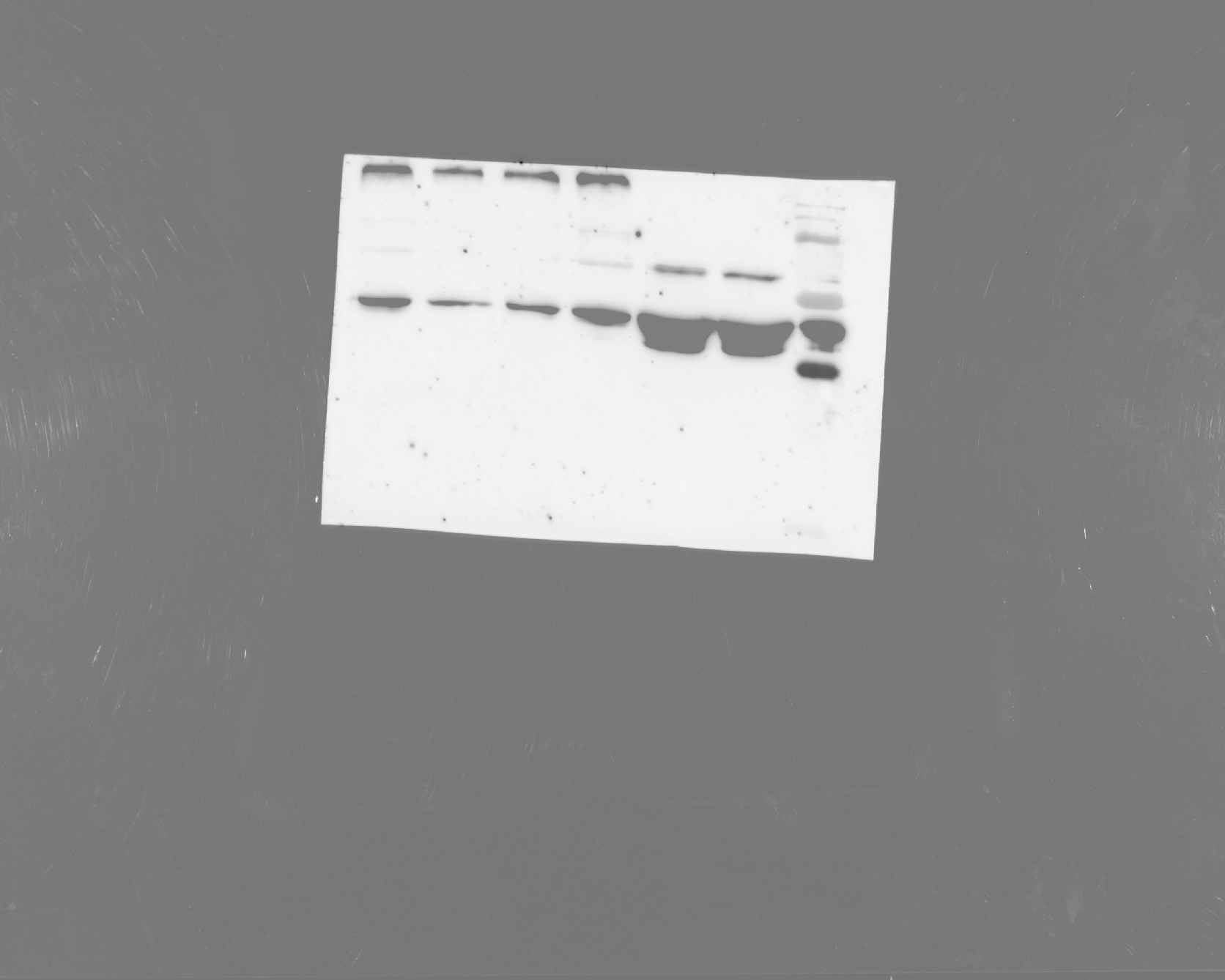

Supplement: Figure 3—source data 2. [file elife-110942-fig3-data2.zip › Figure 3-Source data 2/Figure 3B ii_beta Actin control.jpg]

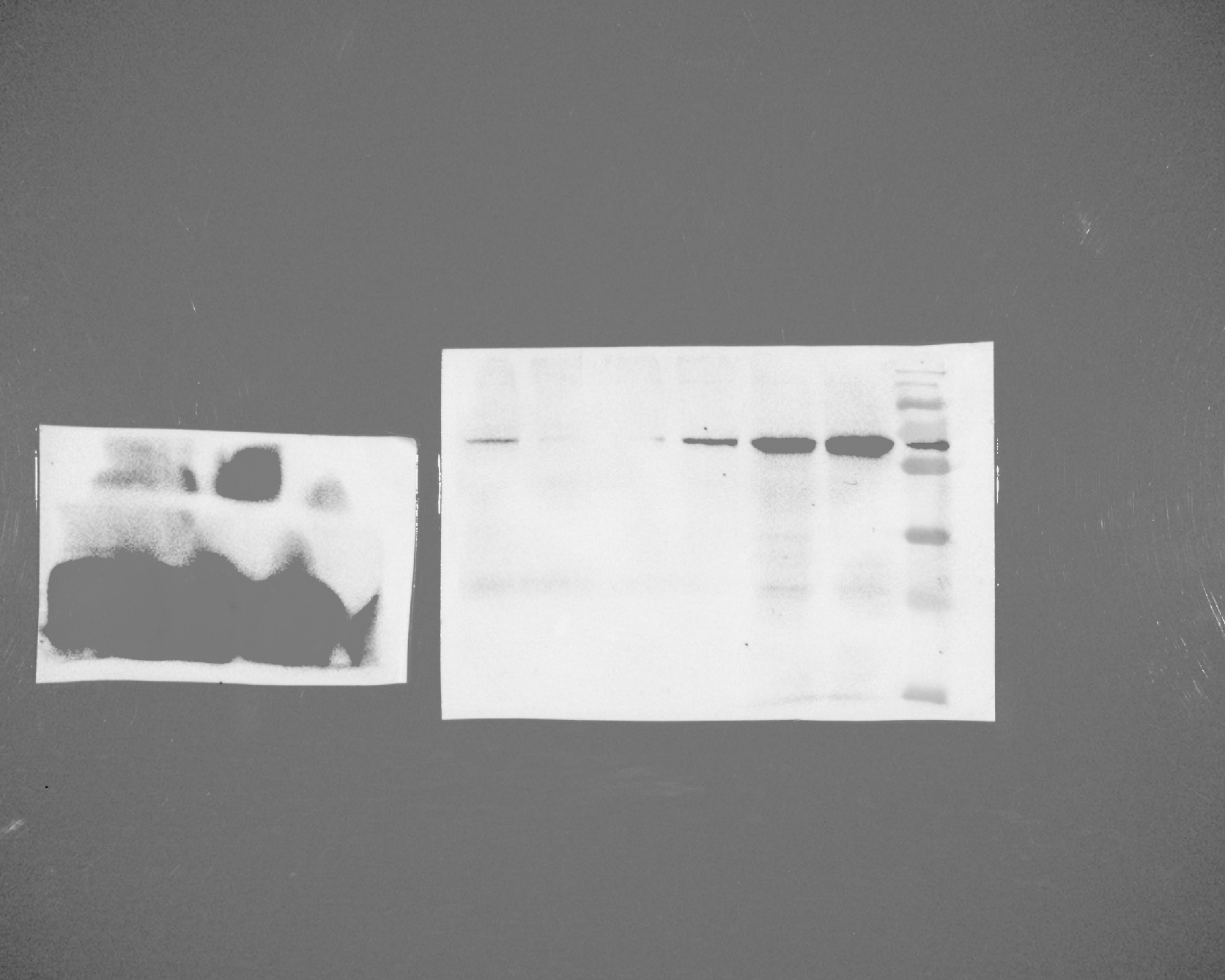

Supplement: Figure 3—source data 2. [file elife-110942-fig3-data2.zip › Figure 3-Source data 2/Figure 3B ii_LFA1.tif]

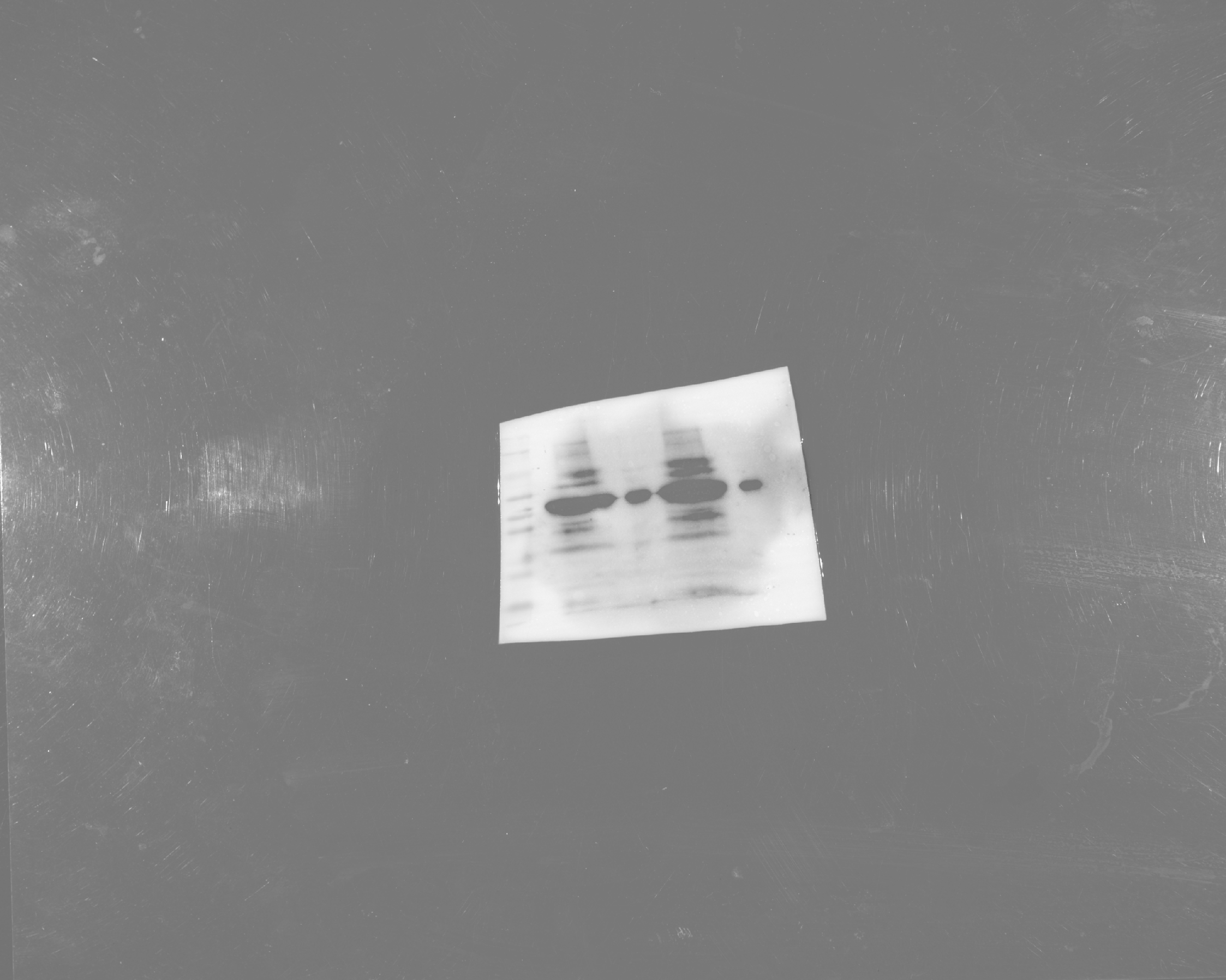

Supplement: Figure 3—source data 2. [file elife-110942-fig3-data2.zip › Figure 3-Source data 2/Figure 3C ii_Beta Actin control.tif]

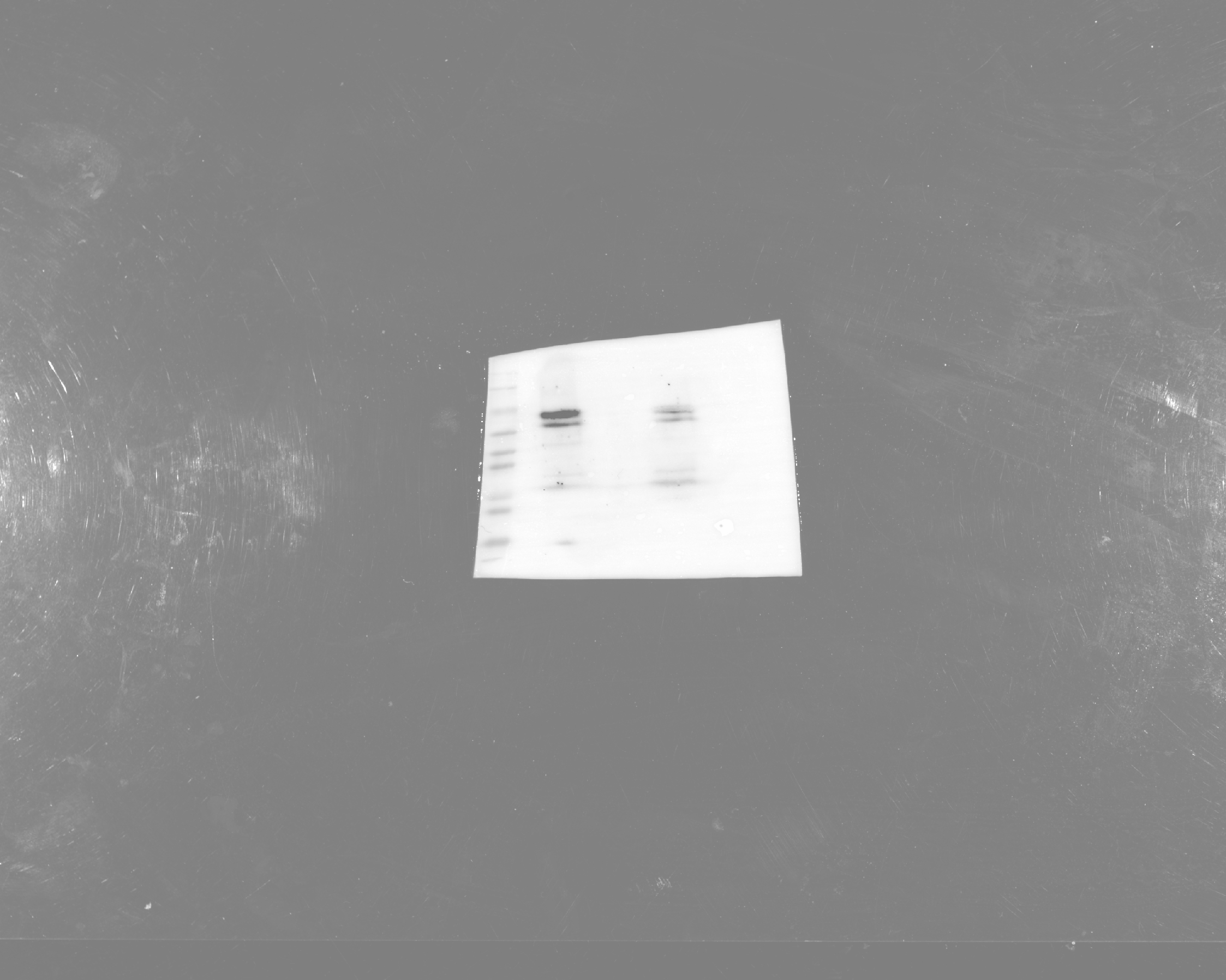

Supplement: Figure 3—source data 2. [file elife-110942-fig3-data2.zip › Figure 3-Source data 2/Figure 3C ii_LFA1.jpg]

Figure 3-figure supplement 1A

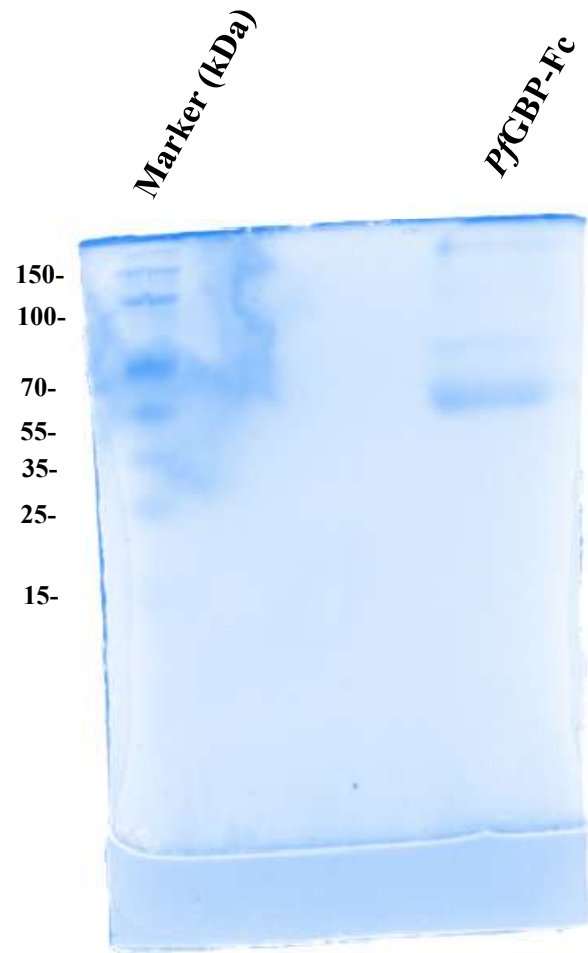

Figure 3-figure supplement 1B

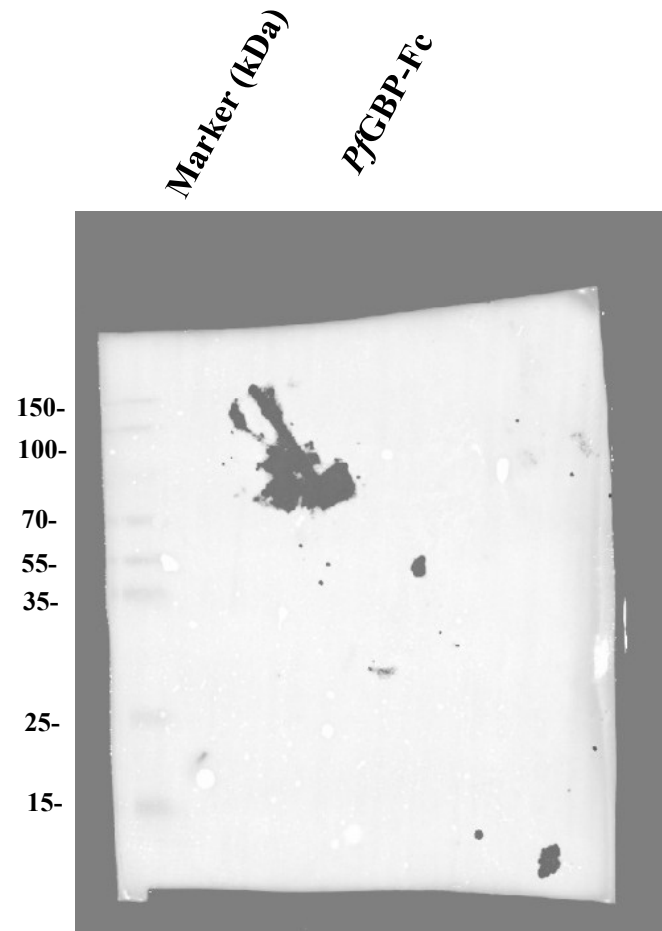

Supplement: Figure 3—figure supplement 1—source data 1. [file elife-110942-fig3-figsupp1-data1.zip › Figure 3-figure supplement 1-Source data 1/PDF of Raw images Figure3-figure supplement 1A & 1B.pdf]

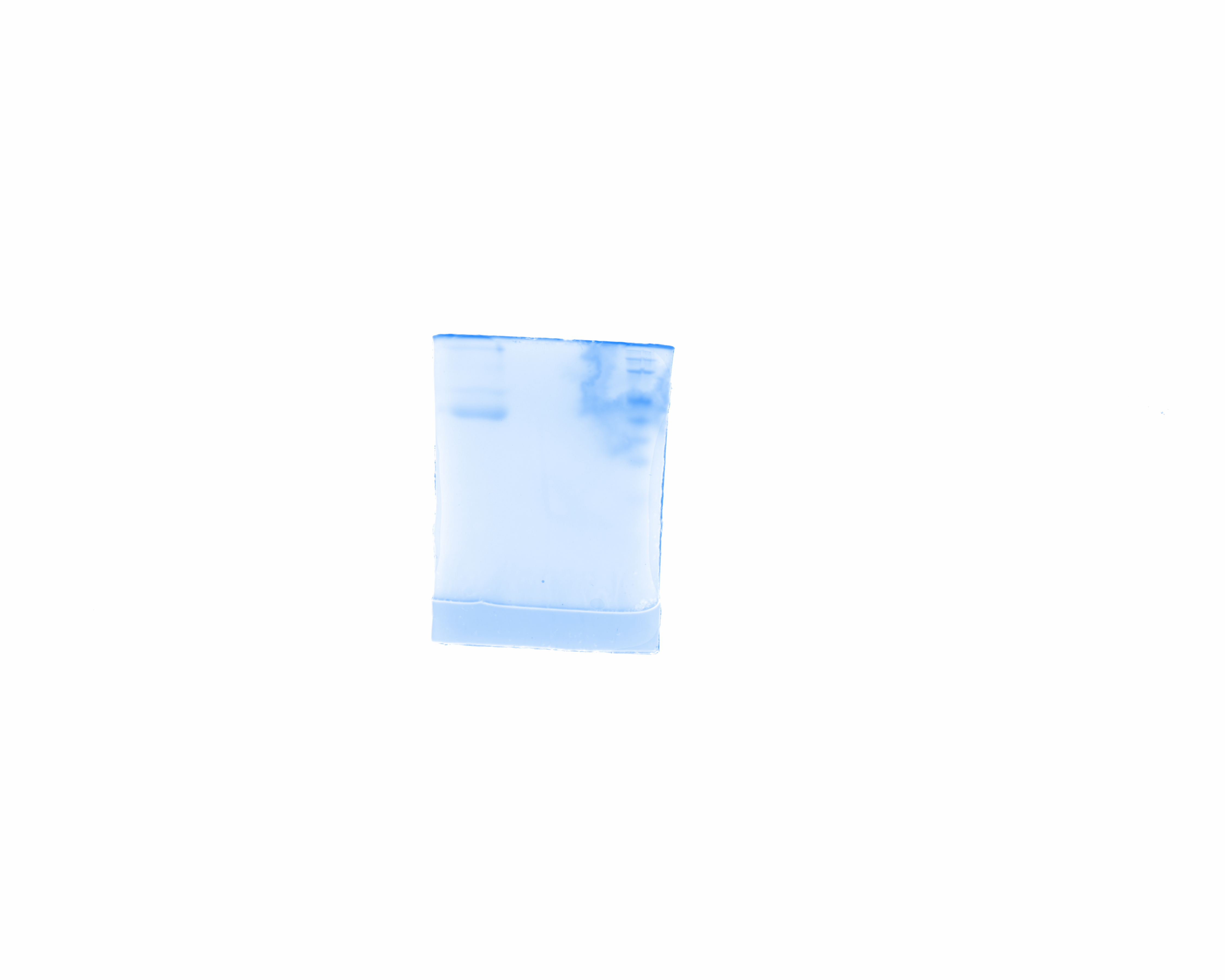

Supplement: Figure 3—figure supplement 1—source data 2. [file elife-110942-fig3-figsupp1-data2.zip › Figure 3-figure supplement- Source data 2/Figure 3-figure supplement 1A.tif]

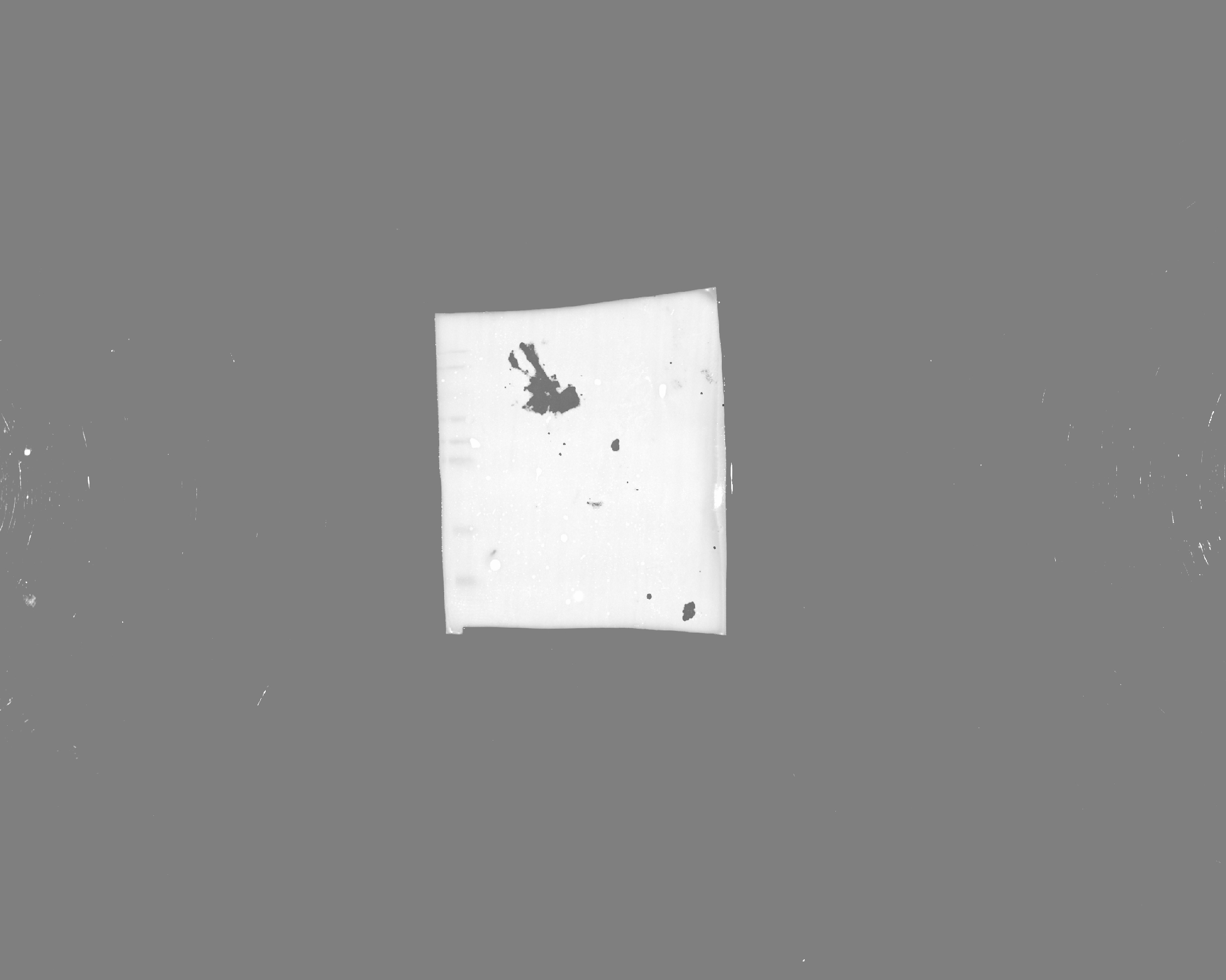

Supplement: Figure 3—figure supplement 1—source data 2. [file elife-110942-fig3-figsupp1-data2.zip › Figure 3-figure supplement- Source data 2/Figure 3-figure supplement 1B.jpg]
