# Supplementary material for: LFA-1 interaction with GBP-130 on Plasmodium falciparum-infected red blood cells mediates NK cell activation and parasite control
Source: eLife. 2026 May 28;15:RP110942. doi: 10.7554/eLife.110942 (PMC13218722; doi:10.7554/eLife.110942)

Figure 2-figure supplement 1B

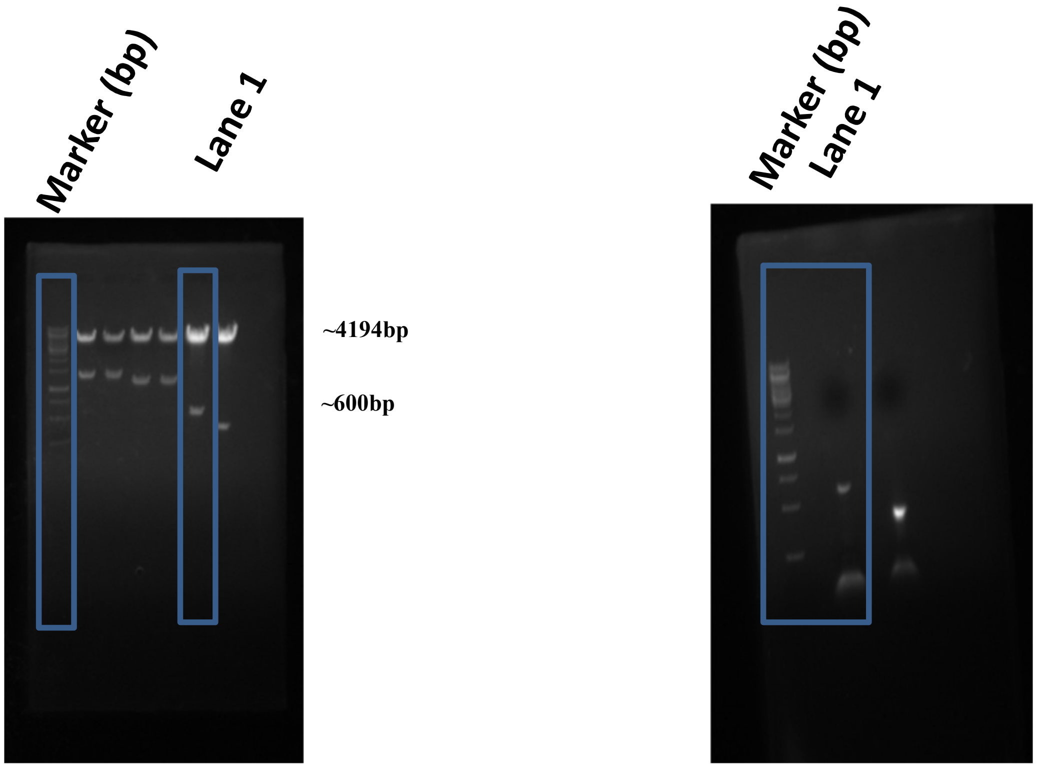

Figure 2-figure supplement 1C_i

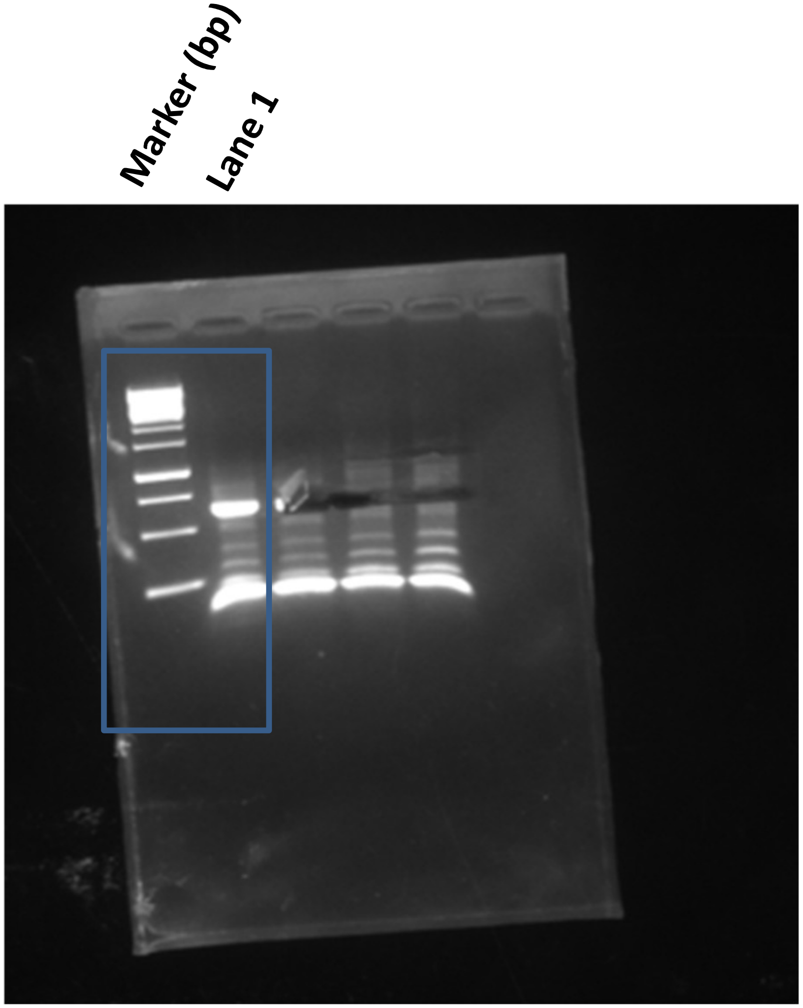

Figure 2-figure supplement 1C_ii

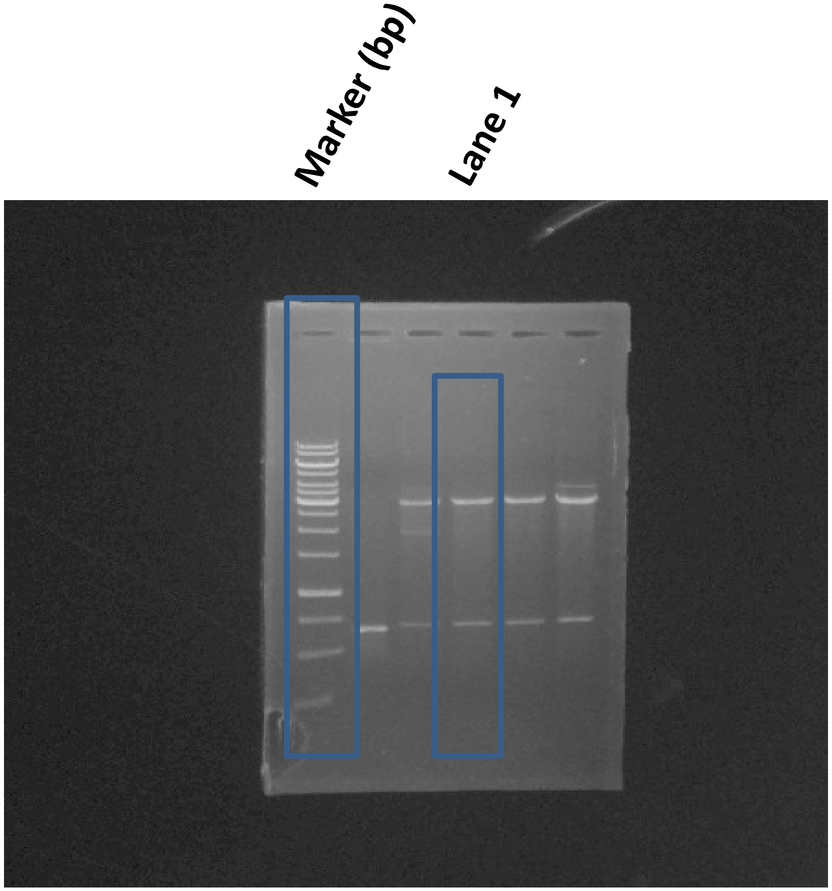

Figure 2-figure supplement 1C_iii

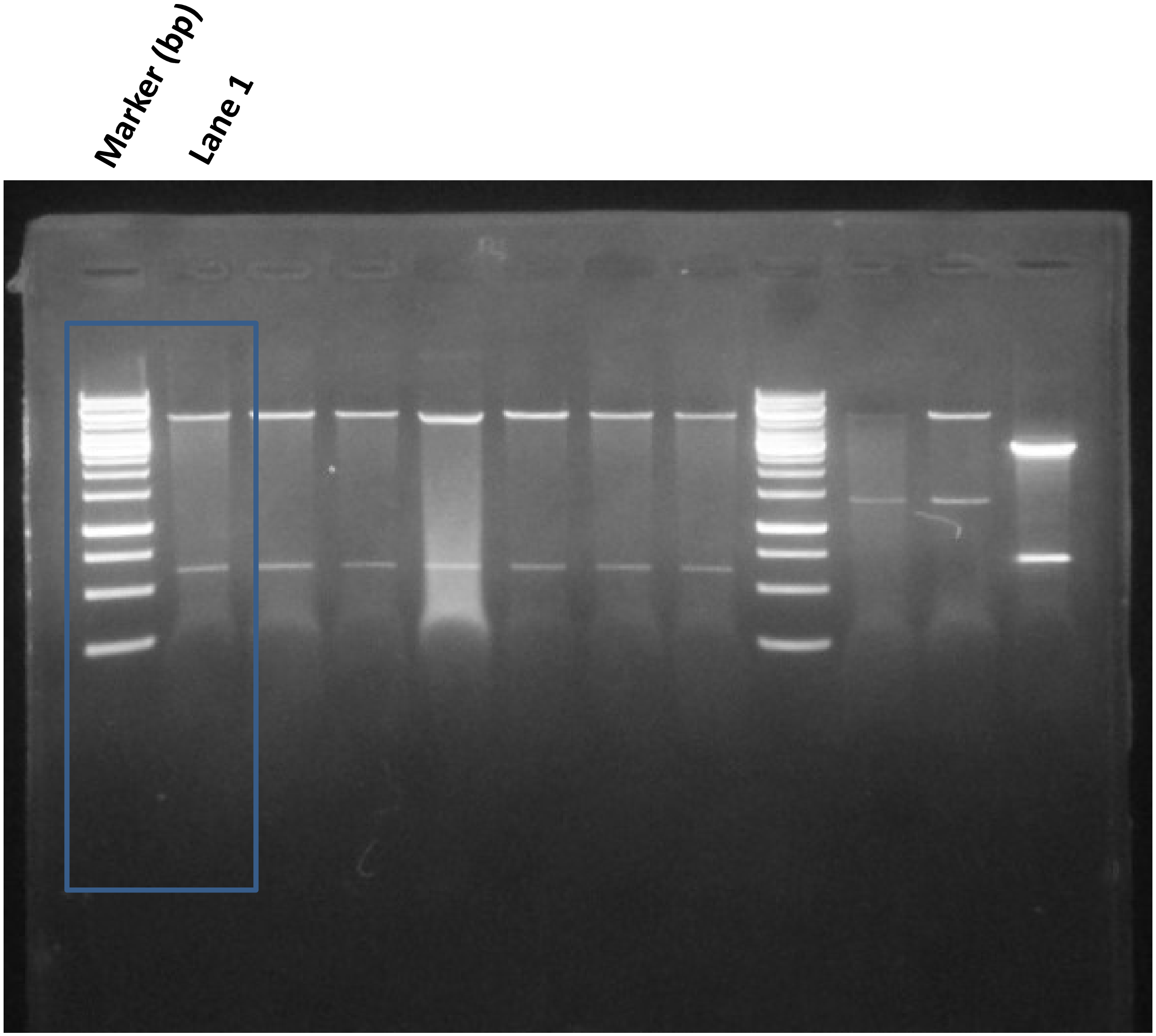

Supplement: Figure 2—figure supplement 1—source data 1. [file elife-110942-fig2-figsupp1-data1.zip › Figure 2-figure supplement 1-Source data 1/PDF of Raw images Figure2-figure supplement 1B & 1C i-iii.pdf]
