# Supplementary material for: LFA-1 interaction with GBP-130 on Plasmodium falciparum-infected red blood cells mediates NK cell activation and parasite control
Source: eLife. 2026 May 28;15:RP110942. doi: 10.7554/eLife.110942 (PMC13218722; doi:10.7554/eLife.110942)
Supplement: Supplementary file 1. — Table of MS/MS hits of the beads+hIgG control. Table B. Docking scores and binding energy calculated using various computational tools to assess the quality of the GBP-LFA1 docked complex. [file elife-110942-supp1.docx]

**Supplementary File 1**

**Table A: Table of MS/MS hits of the beads+hIgG control**

| ***Acession No.*** | ***Description*** | ***Coverage [%]*** | ***# Peptides*** | ***# PSMs*** | ***# Unique Peptides*** | ***# AAs*** | ***MW [kDa]*** | ***calc. pI*** |
| --- | --- | --- | --- | --- | --- | --- | --- | --- |
| PF3D7_0818900 | Heat shock protein 70 | 46 | 34 | 136 | 25 | 677 | 73.9 | 5.67 |
| PF3D7_0917900 | Heat shock protein 70 | 52 | 40 | 113 | 38 | 652 | 72.3 | 5.31 |
| PF3D7_1015900 | Enolase | 58 | 27 | 90 | 26 | 446 | 48.6 | 6.6 |
| PF3D7_0708400 | Heat shock protein 90 | 39 | 37 | 105 | 32 | 745 | 86.1 | 5.01 |
| PF3D7_0930300 | Merozoite surface protein 1 | 28 | 50 | 117 | 50 | 1720 | 195.6 | 6.51 |
| PF3D7_0207600 | Serine-repeat antigen protein 5 | 36 | 33 | 106 | 32 | 997 | 111.7 | 5.41 |
| PF3D7_0929400 | High molecular weight rhoptry protein 2 | 32 | 39 | 92 | 39 | 1378 | 162.6 | 8.27 |
| PF3D7_1357000 | Elongation factor 1-alpha | 58 | 26 | 80 | 26 | 443 | 48.9 | 9.06 |
| PF3D7_1444800 | Fructose-bisphosphate aldolase | 51 | 23 | 75 | 23 | 369 | 40.1 | 8.12 |
| PF3D7_1462800 | Glyceraldehyde-3-phosphate dehydrogenase | 60 | 24 | 70 | 22 | 337 | 36.6 | 7.69 |
| PF3D7_1410400 | Rhoptry-associated protein 1 | 31 | 23 | 65 | 23 | 782 | 90 | 7.11 |
| PF3D7_0922500 | Phosphoglycerate kinase | 67 | 25 | 70 | 25 | 416 | 45.4 | 7.83 |
| PF3D7_0500800 | Mature parasite-infected erythrocyte surface antigen | 29 | 26 | 64 | 26 | 1434 | 168.2 | 4.78 |
| PF3D7_0511800 | Inositol-3-phosphate synthase 1 | 46 | 28 | 66 | 28 | 604 | 69.1 | 7.4 |
| PF3D7_0619400 | Cell division cycle protein 48 homologue, putative | 27 | 21 | 53 | 21 | 828 | 92.3 | 5.08 |
| PF3D7_1451100 | Elongation factor 2 | 26 | 21 | 58 | 21 | 832 | 93.5 | 6.8 |
| PF3D7_0708800 | Heat shock protein 110 | 22 | 16 | 36 | 16 | 873 | 99.9 | 5.69 |
| PF3D7_1324900 | L-lactate dehydrogenase | 42 | 15 | 60 | 15 | 316 | 34.1 | 7.55 |
| PF3D7_1246200 | Actin-1 | 50 | 16 | 44 | 15 | 376 | 41.8 | 5.34 |
| PF3D7_1222300 | Endoplasmin, putative | 28 | 22 | 43 | 22 | 821 | 95 | 5.41 |
| PF3D7_0831700 | Heat shock protein 70 | 31 | 19 | 49 | 9 | 679 | 75 | 5.77 |
| PF3D7_0818200 | 14-3-3 protein I | 48 | 15 | 44 | 14 | 262 | 30.2 | 4.92 |
| PF3D7_1343000 | Phosphoethanolamine N-methyltransferase | 55 | 15 | 53 | 15 | 266 | 31 | 5.6 |
| PF3D7_0626800 | Pyruvate kinase | 30 | 13 | 34 | 13 | 511 | 55.6 | 7.55 |
| PF3D7_0827900 | Protein disulfide-isomerase | 48 | 20 | 48 | 20 | 483 | 55.5 | 5.78 |
| PF3D7_0608800 | Ornithine aminotransferase | 31 | 15 | 45 | 15 | 414 | 46 | 6.89 |
| PF3D7_1436000 | Glucose-6-phosphate isomerase | 30 | 15 | 30 | 15 | 579 | 67.3 | 7.24 |
| PF3D7_1468700 | Eukaryotic initiation factor 4A | 53 | 18 | 43 | 17 | 398 | 45.3 | 5.69 |
| PF3D7_1252100 | Rhoptry neck protein 3 | 12 | 22 | 49 | 22 | 2215 | 263 | 9.2 |
| PF3D7_0524000 | Karyopherin beta | 20 | 19 | 42 | 19 | 1123 | 127.3 | 4.92 |
| PF3D7_0302500 | Cytoadherence linked asexual protein 3.1 | 15 | 20 | 48 | 20 | 1417 | 167.1 | 7.18 |
| PF3D7_1454400 | Aminopeptidase P | 29 | 21 | 41 | 21 | 777 | 90.1 | 6.81 |
| PF3D7_1228600 | Merozoite surface protein 9 | 26 | 14 | 32 | 14 | 743 | 86.6 | 4.86 |
| PF3D7_1361800 | Glideosome-associated connector | 10 | 21 | 44 | 21 | 2605 | 290.8 | 5.25 |
| PF3D7_1105000 | Histone H4 | 53 | 10 | 58 | 9 | 103 | 11.4 | 11.22 |
| PF3D7_1012400 | Hypoxanthine phosphoribosyltransferase | 47 | 11 | 33 | 11 | 231 | 26.3 | 7.71 |
| PF3D7_0922200 | S-adenosylmethionine synthase | 32 | 12 | 31 | 12 | 402 | 44.8 | 6.74 |
| PF3D7_1029600 | Adenosine deaminase | 40 | 15 | 32 | 15 | 367 | 42.4 | 5.69 |
| PF3D7_0915400 | Probable ATP-dependent 6-phosphofructokinase | 17 | 17 | 34 | 17 | 1418 | 159.4 | 6.76 |
| PF3D7_0905400 | High molecular weight rhoptry protein 3 | 12 | 10 | 32 | 10 | 897 | 104.8 | 6.67 |
| PF3D7_1134000 | Heat shock protein 70 | 23 | 12 | 28 | 12 | 663 | 73.3 | 6.84 |
| PF3D7_1015600 | Heat shock protein 60 | 27 | 13 | 28 | 13 | 580 | 62.5 | 7.12 |
| PF3D7_1344200 | Endoplasmic reticulum chaperone GRP170 | 16 | 15 | 33 | 15 | 932 | 108.1 | 5.67 |
| PF3D7_1360800 | Falcilysin | 16 | 15 | 28 | 15 | 1193 | 138.8 | 7.01 |
| PF3D7_0624000 | Hexokinase | 27 | 13 | 36 | 13 | 493 | 55.2 | 7.09 |
| PF3D7_1446200 | M17 Leucyl aminopeptidase | 21 | 11 | 26 | 11 | 605 | 67.8 | 8.6 |
| PF3D7_0501600 | Rhoptry-associated protein 2 | 37 | 12 | 24 | 12 | 398 | 46.7 | 8.79 |
| PF3D7_1008900 | Adenylate kinase 1 | 43 | 10 | 26 | 9 | 242 | 27.6 | 8.85 |
| PF3D7_1224300 | Polyadenylate-binding protein | 13 | 11 | 23 | 11 | 875 | 97.2 | 8.88 |
| PF3D7_1439900 | Triosephosphate isomerase | 38 | 11 | 31 | 11 | 248 | 27.9 | 6.42 |
| PF3D7_1347500 | DNA/RNA-binding protein Alba 4 | 28 | 11 | 23 | 11 | 372 | 42.1 | 7.5 |
| PF3D7_1008700 | Tubulin beta chain | 26 | 10 | 31 | 10 | 445 | 49.7 | 4.83 |
| PF3D7_1120100 | Phosphoglycerate mutase | 37 | 10 | 22 | 10 | 250 | 28.8 | 8.28 |
| PF3D7_1011800 | PRE-binding protein | 11 | 11 | 21 | 11 | 1139 | 131.5 | 9.16 |
| PF3D7_0520900 | Adenosylhomocysteinase | 23 | 11 | 25 | 11 | 479 | 53.8 | 5.92 |
| PF3D7_1124600 | Ethanolamine kinase | 20 | 11 | 22 | 11 | 423 | 49.9 | 5.81 |

**Table B: Docking scores and binding energy calculated using various computational tools to assess the quality of the GBP-LFA1 docked complex.**

| Scores from ClusPro | | | |
| --- | --- | --- | --- |
| Complex Name | **Cluster Member** | **Weighted Score-Center** | **Weighted Score-Lowest Energy** |
| LFA1/*Pf*GBP | 226 | -805.6 | -834.0 |
|  | | | |
| Binding free Energy (kcal/mol) for LFA1/*Pf*GBP complex | | | |
| PRODIGY | -9.8 |  | |
| HawkDock server V2 | -22.2 |  |  |
| PPCheck | -7.6 |  |  |
| AREA-AFFINITY | -10.14 |  |  |
|  | | | |
